# Psychological distress among Ethiopian migrant returnees who were in quarantine in the context of COVID-19: institution-based cross-sectional study

**DOI:** 10.1186/s12888-021-03429-2

**Published:** 2021-08-25

**Authors:** Kassahun Habtamu, Yekoyealem Desie, Mulat Asnake, Endirias Gina Lera, Temesgen Mequanint

**Affiliations:** 1 School of Psychology, College of Education and Behavioral Studies, Addis Ababa University, P.O.BOX: 1176, Addis Ababa, Ethiopia; 2 Research, Consultancy and Community Service Department, Ethiopian Police University, Sendafa, Ethiopia

**Keywords:** Psychological distress, Depression, Anxiety, Stress, Quarantine, COVID-19, Migrant returnees, Ethiopia

## Abstract

**Background:**

In association with the novel coronavirus (SARS-CoV-2) disease 2019 (COVID-19) pandemic, many numbers of Ethiopian migrants are returning to their home country, and they are required to stay in mandatory quarantine centers. This results in severe disruptions of life routines, social isolation, and loss of freedom. Studies on psychological distress among Ethiopian migrant returnees in the context of COVID-19 are scarce. This study aimed to investigate the prevalence of psychological distress and associated factors among migrant returnees who were in quarantine during the time of COVID-19.

**Methods:**

A cross-sectional study was conducted with 405 migrant returnees recruited from quarantine centers in Addis Ababa. We developed a structured questionnaire to collect data on sociodemographic, migration related, quarantine related and COVID-19 related characteristics of participants. We used the 21 item Depression, Anxiety and Stress Scale to assess psychological distress. Univariate and multivariable negative binomial regression models were fitted to assess the association between exposure variables with depression, anxiety and stress separately.

**Results:**

A little more than half of the participants (55%) had depressive symptoms; around half had anxiety symptoms (48.9%) and more than a third (35.6%) experienced symptoms of stress. We found significantly higher prevalence of anxiety (ARR = 0.59; 95% CI = 0.39, 0.91) and depressive symptoms (ARR = 0.56; 95% CI = 0.39, 0.81) among women than men. Fear of discrimination after the quarantine was significantly associated with depressive (ARR = 0.76; 95% CI = 0.63, 0.92) and anxiety symptoms (ARR = 0.77; 95% CI = 0.62, 0.97). Experiencing COVID-19 like symptoms is associated with depressive (ARR = 0.40; 95% CI = 0.25, 0.65), anxiety (ARR = 0.35; 95% CI = 0.20, 0.62) and stress symptoms (ARR = 0.43; 95% CI = 0.28, 0.66). Have no a plan of what to do after the quarantine (ARR = 1.30; 95% CI = 1.09, 1.54) was significantly associated with increasing stress scores.

**Conclusions:**

We found a very high prevalence of depressive, anxiety and stress symptoms among Ethiopian migrant returnees who were in quarantine due to the COVID-19 pandemic. Screening, integration of mental health services with other socioeconomic and psychosocial services, and effective and efficient referral may be useful to address the burden of psychological distress in this group.

**Supplementary Information:**

The online version contains supplementary material available at 10.1186/s12888-021-03429-2.

## Background

Ethiopia is one of the major sources of migrant flows, particularly to the Middle East and South Africa [1]. The number of international Ethiopian migrants is estimated to be one and half million, and each year close to 120, 000 Ethiopians migrate [2]. Migrants from Ethiopia mostly involve in domestic work and other non-professional works [3]; most migrants are young men and women (roughly from early twenties to early thirties) [4]. Ethiopian migrants to the Middle East are mostly women and to South Africa are mostly men [5]. Several studies identified the push and pull factors for migration in Ethiopia, in particular and in Africa in general [2, 3, 6, 7]. Unemployment/underemployment, conflict, poverty, income inequality, household indebtedness, population pressure, lack of good governance, low agricultural productivity and agro-climatic disasters, low wages and insecure informal economic activities are important push factors for people to leave their homes [6, 7]. Attractive working conditions and demand for cheap labor in destinations, the growth of the service industry, aging population in developed countries, booming oil economy in the Middle East and globalization and modernization that have improved communication, transportation and social connection are the important pull factors for migration [8, 9].

Migration, particularly unsafe migration, is found to have impact on migrants physical, psychological, social and economic wellbeing [2, 10]. These include drowning, trafficking, sexual exploitation, labor exploitation, organ harvesting, degrading treatment, discrimination, physical attack and denial of medication [11, 12]. Several qualitative studies among female Ethiopian migrants to the Middle East [8, 13] found experiences of exploitation, enforced cultural isolation, undermining of cultural identity and thwarted expectations. A survey of migrant returnees from the Middle East and South Africa found a high burden of mental health problems resulting from adverse migration experiences, with 27.6% respondents reporting symptoms of common mental disorders [14]. Overall, migrants face a variety of pre-migratory, migratory, and post- migratory stressors that can have lasting impacts on their physical, social and psychological wellbeing [15]. Studies conducted on Ethiopian migrants [13, 16–18] found diverse problems at the various stages of migration. These include physical and emotional abuse while travelling; poor adaptation to the host culture; physical, emotional and sexual abuse at destination; overwork and salary denial; loss of identity and degrading attitudes of employers; very poor legal support; and lack of psychosocial support. These challenges and problems are strongly associated with different kinds of psychosocial problems, including depression, post-traumatic stress disorder and somatization [19].

In relation to the novel coronavirus (SARS-CoV-2) disease 2019 (COVID-19) pandemic, significant numbers of Ethiopian migrant workers from the Middle East and other countries abroad are returning back to their home country. Some of the migrants are returning voluntarily with their own decision, while many others are coming back through mass deportation particularly from Middle East countries of Lebanon, United Arab Emirates, and Saudi Arabia. The deportation is unexpected for the returnees as well as for the Ethiopian government. This mass, sudden and unexpected return as well as the journey during the COVID-19 pandemic may create additional and heightened level of stress, which is likely to be associated with several psychosocial challenges. Migrant returnees are not prepared psychologically and socially for the rapid movement and transitions. They are faced with uncertainty, chaos, personal danger, and complete disruption of normal life sustaining processes [20]. Although global movements are restricted and social distancing is taken as a major protective factor to COVID-19, considerable numbers of Ethiopian migrants from the various countries of the Middle East have been deported to their country. The concerns of migrant returnees to get infected and infecting significant others together with their possible unpreparedness and likely gloomy future may put them at risk of manifesting various psychological and social problems. On top of these challenges, migrant returnees are required to stay in mandatory quarantine centers for a week or two.

Quarantine is often an unpleasant experience for those who undergo it [21]. There might be severe disruptions of routines, separation from family and friends, loss of freedom, uncertainty over disease status, fear of infection, boredom, shortages of food and medicine, wage loss, and social isolation [21, 22]. Studies found a high prevalence of psychological distress in those who have been quarantined, with some experiencing anxiety for their safety or anger about being involuntarily confined [21, 22]. In addition, thinking to start a new life in the home country after quarantine may be gloomy and difficult. Hence, migrant returnees who experienced quarantine are likely to be worried about reintegrating with the community and their family.

A number of qualitative studies [2, 13, 23] were conducted on the social and psychological problems of Ethiopian migrant returnees, particularly from Middle East countries. A few quantitative surveys [14] also investigated the prevalence and associated factors of psychological distress and disorders among this population group. There are a few studies which investigated the impact of COVID-19 on the general population and particularly to healthcare providers [24, 25]. For instance, a web-based cross-sectional survey in Italy found that COVID-19 pandemic has physical and psychological pressure on general practitioners [26]. Nevertheless, to the best of our knowledge, there are no published studies on the prevalence and associated factors of psychological distress among migrant returnees who were in quarantine in the context of COVID-19. This study, therefore, aimed at investigating the prevalence of psychological distress and its association with sociodemographic, and migration, quarantine and COVID-19 related characteristics among migrant returnees who were in quarantine in Addis Ababa, Ethiopia.

## Methods

A cross-sectional survey study was conducted. The study attempted to determine the prevalence and associated factors of psychological distress (depression, anxiety and stress) among an institution-based sample of migrant returnees who were in quarantine during the time of COVID-19. The study was conducted from 1st May to 15 June 2020.

### Study setting and context

The study was conducted in quarantine centers in Addis Ababa, Ethiopia. After the identification of the first few COVID-19 cases in Ethiopia (on approximately March 4th, 2020), the government took several measures, one of which is establishing quarantine centers for individuals coming from abroad, those tested positive for the virus and those who had contact with persons who have COVID-19. Quarantine centers in Addis Ababa were established in hospitals, primary healthcare centers, schools, university campuses and convention centers.

The current study was conducted in seven quarantine centers which were established in three universities in Addis Ababa. Our qualitative exploration showed that the quarantine centers had basic facilities (a separate room for each person, bed, blanket, and shared toilet and shower). There were no complaints over food, water and other basic necessities. During the time of this study, migrant returnees were required to stay in the quarantine center for 14 days. Security forces were available to prevent participants from leaving the center, and communicating in person or having physical contact with others. However, they were allowed to make and receive telephone calls.

### Participants and sampling

We selected seven quarantine centers in Addis Ababa using convenience sampling as they were near and accessible to the research team. Five centers (the Main Campus, College of Business and Economics Campus, College of Natural Sciences Campus, Lideta Campus, and Technology Campus) from Addis Ababa University, one center from Addis Ababa Science and Technology University, and another center from Ethiopian Civil Service University were selected for the study. In these seven quarantine centers, there were around 6500 migrant returnees during the time of this study (2850 in the different quarantine centers in Addis Ababa University, 3060 in Addis Ababa Science and Technology University Center and 590 in Ethiopian Civil Service University Center).

We invited 416 migrant returnees to participate (182 from Addis Ababa University Centers, 38 from Ethiopian Civil Service University Center and 196 from Addis Ababa Science and Technology University Center). The inclusion criteria were being an Ethiopian migrant returnee during the time of COVID-19, stayed in one of the seven quarantine centers for at least ten days, being an adult (age 18 years or older), able to answer the survey questions in Amharic and able to give verbal informed consent. Those who have a known severe mental illness or who have acute physical illness were excluded from the study. Of those invited to participate, 405 agreed and completed the survey (a response rate of 97.4%).

### Assessment of predictor variables

We developed a structured questionnaire to collect data on sociodemographic, migration related, quarantine related and COVID-19 related characteristics of participants (see, additional file 1). The questionnaire included several closed-ended questions related to sociodemographic characteristics (four questions), returnees’ experiences in pre-migration, while traveling and in the destination (five questions), questions related to returnees’ quarantine experiences (10 questions) and COVID-19 related characteristics (three questions). The questionnaire was reviewed by experts who have experience in scale development, adaption and validation. One of the team members has rich experience to develop, adapt and validate psychometric scales. All of the team members have training and experience in qualitative and quantitative research. The experts who reviewed the questionnaire have also research experience in the area of migration and health. We pilot tested the questionnaire with respondents having similar attributes as the main study participants. The same inclusion criteria as the main study were considered when selecting the pilot participants. Based on the findings of the pilot study, we modified questions which were less understandable, sensitive and less acceptable.

### Assessment of outcome variables

We used the 21 item Depression Anxiety and Stress Scale (DASS21) to assess psychological distress [27]. Depression, Anxiety and Stress Scale (DASS) is one of the commonly-used scales for measuring psychological distress. It is developed by Lovibond and Lovibond to assess symptoms of depression, anxiety and stress among adults [28]. The DASS21 is the shortened version of the DASS. Since its introduction in 1995, the DASS and its short-form the DASS21 have been widely used to assess depression, anxiety and stress among adults [29]. There are 21 items in this scale with four response options: 0 (Did not apply to me at all), 1 (Applied to me to some degree), 2 (Applied to me to a considerable degree), 3 (Applied to me very much, or most of the time) [30]. Each of the three sub-scales (depression, anxiety and stress) has seven items. Scores on the three subscales can then be calculated, which ranges from 0 to 21.

Several studies on the psychometric properties of this measure yielded consistent results [29, 31–34]. The reliability and validity of both the DASS and the DASS21 have been replicated among clinical, as well as non-clinical adult samples [32, 35]. The three-factor structure of the scale is consistent when it is used in diverse cultures and contexts [36, 37]. Scores on the DASS21 are found to have very high correlation with scores on other measures of depression, anxiety and stress (e.g. the Beck Depression Inventory, the Beck Anxiety Inventory and the State-Trait Anxiety Inventory) [32]. The DASS21is also found to be a valid and reliable instrument for measuring depression, anxiety, and stress in the workplace in the African context [38]. For DASS21 sub-scale severity ratings, the cut-off points in Table 1 below are suggested [39].

**Table 1:** DASS21 sub-scale severity ratings

Severity	Depression (DASS21-D)	Anxiety (DASS21-A)	Stress (DASS21-S)
Normal	0–4	0–3	0–7
Mild	5–6	4–5	8–9
Moderate	7–10	6–7	10–12
Severe	11–13	8–9	13–16
Extremely sever	14+	10+	17+

DASS21 = The 21 item Depression Anxiety and Stress Scale.

The Amharic version of the DASS21 has been used in previous published studies in Ethiopia [40, 41]. For this study, the original version of DASS21 has been translated and back-translated by four members of the research team who are fluent Amharic speakers and trained at masters’ or PhD degree level following standard procedures. Senior members of the research team, who have training and experience in scale adaptation and validation, evaluated the relevance, cultural equivalence, acceptability and clarity of each item of the Amharic version of the scale. The Amharic version of the DASS21 has been used previously in Ethiopia; however, it has not been validated in the Ethiopian socio-cultural context. We pilot tested the measure with respondents having similar attributes as the main study participants. Based on the findings of the pilot study, we modified items or translations which were less understandable, sensitive and less acceptable.

### Data collection procedure

The process of data collection was executed by two master’s degree level trained and experienced members of the research team. Both of the field coordinators were members of the research team and they have master’s degree in psychology. These members of the research team were also in charge of coordinating and supervising the quarantine centers employed by the Ethiopian Federal Ministry of Peace and Ministry of Health. The other three senior members of the research team supervised and coordinated the data collection process. The senior members of the research team trained those who executed the data collection, oversee participant recruitment and data collection and involve in checking and controlling data quality. Half- day orientation was provided for those who executed the data collection on the purpose of the study, the contents of the data collection instruments, ethical matters, and on how to recruit and approach participants.

Data collection was done in quarantine centers (house-to-house) where migrant returnees were available via the guidance of key informants. Data collectors provided the questionnaire to those who gave consent and collected back the completed questionnaires after three days. Data collectors interviewed those who are not able to read and write. The senior members of the research team closely followed-up the data collection process.

### Data management and analysis

Data were entered into Statistical Packages for the Social Sciences (SPSS version 24) by trained and experienced data entry clerks and then exported into STATA for windows (version 13) for analysis. The completeness and consistency of the data collected by field coordinators was checked by the first author prior to data analyses. Frequencies and percentages were used to summarize variables which were categorical. Continuous variables were summarized using mean and standard deviation.

Descriptive statistics were used to determine the prevalence of psychological distress (depression, anxiety and stress). Univariate and multivariable negative binomial regression models were fitted to assess the association of sociodemographic, migration related, quarantine related and COVID-19 related characteristics with depression, anxiety and stress separately. Factors that were associated with the outcome variables (depression, anxiety and stress) in the univariate models with *P*-value < 0.2 were included in the corresponding multivariable models in order to limit the potential risk of over adjusting without compromising identification of potential predictors for the outcome variables. Relative risk (RR), both crude and adjusted, with the corresponding 95% confidence interval, was used to estimate the strength of association between potential associated factors and the outcome variables in both the univariate and the multivariable models. All statistical tests were set at α = 0.05 for significance.

### Ethical considerations

The study protocol was reviewed and approved by a technical committee established by the Office of the Vice President for Research and Technology Transfer (VPRTT) at Addis Ababa University. We secured a support letter from the VPRTT to collect data from the quarantine centers. We obtained permission to collect data from the coordinators of the quarantine centers by presenting a cooperation letter written from Addis Ababa University. All methods related to the human participants were performed in accordance with the Declaration of Helsinki. Participation was voluntary and verbal informed consent was obtained from all the participants after the nature of the study was fully explained to them. We preferred verbal informed consent to written informed consent just to put respondents at ease since informants may not be comfortable to put their signature on paper in the Ethiopian socio-cultural context. Respondents were explicitly informed that they could withdraw at any time from the study and cease to respond to any question they felt uncomfortable. Information obtained from all the participants was anonymized and confidentiality was assured throughout the data collection process.

## Results

### Characteristics of the participants

A total of 405 migrant returnees, who were in quarantine, participated in the study. The majority of the participants were women (92.3%), with average age 25.8 years (SD = 3.58). Around 70% of the returnees were single and less than a quarter currently married (22.7%). More than half of the participants had either secondary (55.3%) or post-secondary (8.1%) level of education. Details of the characteristics of the participants are presented in Table 2.

**Table 2:** Sociodemographic, migration, quarantine and COVID-19 related characteristics of participants (*n* = 405)

Characteristics		Number	Percent
**Socio-demographic characteristics**			
Gender	Female	374	92.3
	Male	31	7.7
Age (in years)	Mean (SD)	25.8 (3.58)	
Education	Cannot read and write	23	5.7
	Primary school	125	30.9
	Secondary school	224	55.3
	Post-secondary	33	8.1
Marital status	Never married	284	70.1
	Currently married	92	22.7
	Previously married	29	7.2
**Migration related characteristics**			
Status in the host country before return	On job	264	65.2
	Detention center	66	16.3
	Prison	43	10.6
	Unemployed	32	7.9
Host (destination) country	Jordan	293	72.3
	United Arab Emirates	49	12.1
	Qatar	26	6.4
	Iran	12	3.0
	Bahrain	8	2.0
	Saudi Arabia	2	0.5
	Ukraine	8	2.0
	Australia	4	1.0
	Spain	3	0.7
How did you go to the destination country? (way of migration)	Through travel agency	331	81.7
	Through broker	74	18.3
Underlying physical health problem	Yes	20	4.9
	No	385	95.1
Underlying mental health problem	Yes	8	2.0
	No	397	98.0
**Quarantine related characteristics**			
Fear of infection in quarantine center	Yes, I was afraid	159	39.3
	No, I was not afraid	246	60.7
Staying in quarantine protected me not to transmit the virus to family and community	Yes	361	89.1
	No	44	10.9
Quarantine limited my activities and social interaction	Yes	337	83.2
	No	68	16.8
Overall, services in the quarantine center was satisfactory	Yes	216	53.3
	No	189	46.7
I know the reason why I am here in quarantine	Yes, I know	392	96.8
	No, I don’t	13	3.2
I got sufficient information about the quarantine from the concerned body	Yes	321	79.3
	No	84	20.7
Fear of discrimination after the quarantine	Yes	177	43.7
	No	228	56.3
I can get support from family and relatives after the quarantine	Yes, I can	245	60.5
	No, I can not	160	39.5
I have a plan of what to do after the quarantine	Yes	146	36.0
	No	259	64.0
I have sufficient amount of money for my living and startup business after the quarantine	Yes	60	14.8
	No	345	85.2
**COVID-19 related characteristics**			
I have adequate knowledge about the mode of transmission and prevention of COVID-19	Yes, I have	341	84.2
	No, I have not	64	15.8
I experienced headache, sore throat, breathing difficulty during my stay in quarantine	Yes	15	3.7
	No	390	96.3
I had contact with a COVID-19 suspected or infected person or I was exposed to situations before the quarantine	Yes, I had	10	2.5
	No, I had not	395	97.5

A little more than a third of the participants (34.8%) were not on job in the host country before return, and the majority (72.3%) came from Jordan. More than 80% of the participants reported that the quarantine experience limited their activities and social interaction and 43.7% of them had fear of discrimination after the quarantine. While more than a third (36.0%) of the returnees had a plan of what to do after the quarantine, only 14.8% had sufficient amount of money for their living and to start their own business after the quarantine. The majority of the participants reported that they have adequate knowledge about the mode of transmission and prevention of COVID-19, and only a few participants had contact with a COVID 19 suspected or infected person (2.5%) and experienced COVID-19 symptoms (headache, sore throat, and breathing difficulty) during their stay in quarantine (3.7%).

### The levels of psychological distress among migrant returnees who were in quarantine

More than half of migrant returnees who were in quarantine (55%) had depressive symptoms (Table 3). Of these, 22.0% had severe (10.1%) or extremely severe (11.9%) symptoms of depression; whereas 32.8% had mild (16.8%) or moderate (16.0%) symptoms of depression. Around half of the participants had anxiety symptoms (48.9%). While 26.1% had severe (8.6%) or extremely severe (17.5%) anxiety symptoms, 22.7% had mild (16.0%) or moderate (6.7%) symptoms of anxiety. More than a third (35.6%) of the returnees experienced symptoms of stress. Of these, 13.4% experienced severe (7.2%) or extremely severe (6.2%) stress, whereas 22.1% experienced mild (10.4%) or moderate (11.7%) stress.

**Table 3:** **Psychological distress levels of participants**

Variable	Frequency	Percent
Depression (*n* = 404)		
Normal (0–4)	182	45.0
Mild (5–6)	68	16.8
Moderate (7–10)	65	16.0
Severe (11–13)	41	10.1
Extremely severe (≥14)	48	11.9
Anxiety (n = 405)		
Normal (0–3)	207	51.1
Mild (4–5)	65	16.0
Moderate (6–7)	27	6.7
Severe (8–9)	35	8.6
Extremely severe (≥10)	71	17.5
Stress (n = 405)		
Normal (0–7)	259	64.4
Mild (8–9)	42	10.4
Moderate (10–12)	47	11.7
Severe (13–16)	29	7.2
Extremely severe (≥17)	25	6.2

COVID-19 = Coronavirus Disease 2019; SD = Standard deviation.

*n = number of participants.*

### Factors associated with depressive, anxiety and stress symptoms

Results from both the univariate and multivariable negative binomial regression models of depression, anxiety and stress scores are presented in Tables 4, 5 and 6. In the univariate models, female gender; migration through broker; fear of infection in the quarantine center; lack of sufficient information about the quarantine; fear of discrimination after the quarantine and experienced headache, sore throat and breathing difficulty during stay in the quarantine center were significantly associated with increasing depressive symptoms. In the adjusted model, male gender (ARR = 0.56; 95% CI = 0.39, 0.81), have no fear of infection in the quarantine center (ARR = 0.80; 95% CI = 0.65, 0.96), have no fear of discrimination after the quarantine (ARR = 0.76; 95% CI = 0.63, 0.92), and have not experienced headache, sore throat, breathing difficulty during stay in quarantine (ARR = 0.40; 95% CI = 0.25, 0.65) were significantly associated with decreasing depressive symptoms.

**Table 4:** Factors associated with depressive symptoms

Variable		CRR (95% CI)	ARR (95% CI)
Gender	Female	1.0	1.0
	Male	**0.51 [0.35, 0.73]**	**0.56 [0.39, 0.81]**
Age (in years)		0.99 [0.96, 1.01]	
Education	Not literate	1.0	
	Primary school	1.02 [0.66, 1.57]	
	Secondary school	1.10 [0.73, 1.67]	
	Post-secondary	1.03 [0.62, 1.72]	
Marital status	Never married	1.0	1.0
	Currently married	0.86 [0.69, 1.08]	0.89 [0.72. 1.11]
	Previously married	1.08 [0.75, 1.55]	1.05 [0.74, 1.47]
Status in the host country before return	On job	1.0	
	Detention center	0.89 [0.69, 1.16]	
	Prison	1.01 [0.75, 1.38]	
	Unemployed	1.03 [0.73, 1.46]	
How did you go to the destination country? (way of migration)	Through travel agency	1.0	1.0
	Through broker	**1.28 [1.01, 1.62]**	1.13 [0.91, 1.42]
Underlying physical health problem	Yes	1.0	
	No	0.76 [0.50, 1.16]	
Underlying mental health problem	Yes	1.0	
	No	1.15 [0.58, 2.27]	
Fear of infection in quarantine center	Yes, I was afraid	1.0	1.0
	No, I was not afraid	**0.78 [0.65, 0.94]**	**0.80 [0.65, 0.96]**
Staying in quarantine protected me not to transmit the virus to family and community	Yes	1.0	
	No	1.03 [0.76, 1.39]	
Quarantine limited my activities and social interaction	Yes	1.0	1.0
	No	0.83 [0.64, 1.06]	0.92 [0.72, 1.17]
Overall, services in the quarantine center was satisfactory	Yes	1.0	
	No	1.07 [0.89, 1.29]	
I know the reason why I am here in quarantine	Yes, I know	1.0	
	No, I don’t	1.23 [0.73, 2.07]	
I got sufficient information about the quarantine from the concerned body	Yes	1.0	1.0
	No	**1.26 [1.00, 1.58]**	1.11 [0.88, 1.39]
Fear of discrimination after the quarantine	Yes	1.0	
	No	**0.73 [0.61, 0.89]**	**0.76 [0.63, 0.92]**
I can get support from family and relatives after the quarantine	Yes, I have	1.0	
	No, I have not	1.01 [0.91, 1.33]	
I have a plan of what to do after the quarantine	Yes	1.0	
	No	1.04 [0.86, 1.27]	
I have sufficient amount of money for my living and startup business after the quarantine	Yes	1.0	
	No	1.08 [0.83, 1.41]	
I have adequate knowledge about the mode of transmission and prevention of COVID-19	Yes, I have	1.0	
	No, I have not	1.28 [0.995, 1.65]	1.15 [0.89, 1.49]
I experienced headache, sore throat, breathing difficulty during my stay in quarantine	Yes	1.0	1.0
	No	**0.42 [0.26, 0.66]**	**0.40 [0.25, 0.65]**
I had contact with a COVID-19 suspected or infected person or I was exposed to situations before the quarantine	Yes, I had	1.0	
	No, I had not	0.79 [0.44, 1.43]

**Table 5:** Factors associated with anxiety symptoms

Variable		CRR (95% CI)	ARR (95% CI)
Gender	Female	1.0	1.0
	Male	**0.58 [0.37, 0.89]**	**0.59 [0.39, 0.91]**
Age (in years)		0.99 [0.96, 1.02]	
Education	Not literate	1.0	
	Primary school	1.03 [0.62, 1.70]	
	Secondary school	0.97 [0.60, 1.58]	
	Post-secondary	0.82 [0.45, 1.50]	
Marital status	Never married	1.0	
	Currently married	1.01 [0.78, 1.32]	
	Previously married	0.95 [0.62, 1.47]	
Status in the host country before return	On job	1.0	
	Detention center	0.97 [0.71, 1.31]	
	Prison	0.95 [0.66, 1.38]	
	Unemployed	0.95 [0.63, 1.44]	
How did you go to the destination country? (way of migration)	Through travel agency	1.0	
	Through broker	1.20 [0.90, 1.59]	
Underlying physical health problem	Yes	1.0	1.0
	No	0.67 [0.41, 1.10]	0.77 [0.48, 1.24]
Underlying mental health problem	Yes	1.0	
	No	0.96 [0.44, 2.12]	
Fear of infection in quarantine center	Yes, I was afraid	1.0	1.0
	No, I was not afraid	0.81 [0.65, 1.02]	0.84 [0.66, 1.01]
Staying in quarantine protected me not to transmit the virus to family and community	Yes	1.0	1.0
	No	0.72 [0.50, 1.03]	0.74 [0.52, 1.07]
Quarantine limited my activities and social interaction	Yes	1.0	1.0
	No	0.82 [0.61, 1.10]	0.92 [0.69, 1.23]
Overall, services in the quarantine center was satisfactory	Yes	1.0	
	No	1.14 [0.92, 1.43]	
I know the reason why I am here in quarantine	Yes, I know	1.0	
	No, I don’t	1.05 [0.56, 1.96]	
I got sufficient information about the quarantine from the concerned body	Yes	1.0	1.0
	No	1.31 [0.999, 1.71]	1.11 [0.84, 1.47]
Fear of discrimination after the quarantine	Yes	1.0	1.0
	No	**0.79 [0.63, 0.90]**	**0.77 [0.62, 0.97]**
I can get support from family and relatives after the quarantine	Yes, I have	1.0	
	No, I have not	0.97 [0.77, 1.21]	
I have a plan of what to do after the quarantine	Yes	1.0	
	No	1.10 [0.87, 1.38]	
I have sufficient amount of money for my living and startup business after the quarantine	Yes	1.0	
	No	0.87 [0.64, 1.18]	
I have adequate knowledge about the mode of transmission and prevention of COVID-19	Yes, I have	1.0	1.0
	No, I have not	1.22 [0.90, 1.64]	1.04 [0.76, 1.43]
I experienced headache, sore throat, breathing difficulty during my stay in quarantine	Yes	1.0	1.0
	No	**0.35 [0.20, 0.59]**	**0.35 [0.20, 0.62]**
I had contact with a COVID-19 suspected or infected person or I was exposed to situations exposed before the quarantine	Yes, I had	1.0	
	No, I had not	0.68 [0.34, 1.35]

**Table 6:** Factors associated with stress

Variable		CRR (95% CI)	ARR (95% CI)
Gender	Female	1.0	1.0
	Male	0.73 [0.53, 1.02]	0.81 [0.57, 1.14]
Age (in years)		0.98 [0.96, 1.01]	0.99 [0.97, 1.01]
Education	Not literate	1.0	
	Primary school	1.07 [0.72, 1.58]	
	Secondary school	1.14 [0.78, 1.66]	
	Post-secondary	0.90 [0.56, 1.43]	
Marital status	Never married	1.0	
	Currently married	0.95 [0.78, 1.17]	
	Previously married	0.95 [0.68, 1.33]	
Status in the host country before return	On job	1.0	
	Detention center	0.94 [0.74, 1.20]	
	Prison	0.94 [0.71, 1.24]	
	Unemployed	1.04 [0.75, 1.44]	
How did you go to the destination country? (way of migration)	Through travel agency	1.0	1.0
	Through broker	1.20 [0.97, 1.50]	1.12 [0.91, 1.39]
Underlying physical health problem	Yes	1.0	
	No	0.86 [0.58, 1.26]	
Underlying mental health problem	Yes	1.0	
	No	1.06 [0.57, 1.96]	
Fear of infection in quarantine center	Yes, I was afraid	1.0	
	No, I was not afraid	0.89 [0.75, 1.06]	
Staying in quarantine protected me not to transmit the virus to family and community	Yes	1.0	
	No	0.88 [0.67, 1.16]	
Quarantine limited my activities and social interaction	Yes	1.0	
	No	0.97 [0.77, 1.22]	
Overall, services in the quarantine center was satisfactory	Yes	1.0	
	No	1.02 [0.86, 1.21]	
I know the reason why I am here in quarantine	Yes, I know	1.0	
	No, I don’t	1.23 [0.77, 1.97]	
I got sufficient information about the quarantine from the concerned body	Yes	1.0	1.0
	No	**1.23 [1.001, 1.51]**	1.14 [0.92, 1.40]
Fear of discrimination after the quarantine	Yes	1.0	1.0
	No	0.89 [0.75, 1.06]	0.85 [0.72, 1.01]
I can get support from family and relatives after the quarantine	Yes, I have	1.0	
	No, I have not	1.04 [0.88, 1.24]	
I have a plan of what to do after the quarantine	Yes	1.0	1.0
	No	**1.21 [1.01, 1.44]**	**1.30 [1.09, 1.54]**
I have sufficient amount of money for my living and startup business after the quarantine	Yes	1.0	
	No	1.01 [0.79, 1.28]	
I have adequate knowledge about the mode of transmission and prevention of COVID-19	Yes, I have	1.0	
	No, I have not	0.96 [0.76, 1.21]	
I experienced headache, sore throat, breathing difficulty during my stay in quarantine	Yes	1.0	1.0
	No	**0.45 [0.30, 0.68]**	**0.43 [0.28, 0.66]**
I had contact with a COVID-19 suspected or infected person or I was exposed to situations before the quarantine	Yes, I had	1.0	
	No, I had not	0.91 [0.53, 1.57]	

In the univariate models, we found female gender; fear of discrimination after the quarantine; and experienced headache, sore throat and breathing difficulty during stay in the quarantine center significantly associated with increasing anxiety symptoms. In the multivariable model, male gender (ARR = 0.59; 95% CI = 0.39, 0.91), have no fear of discrimination after the quarantine (ARR = 0.77; 95% CI = 0.62, 0.97), and have not experienced headache, sore throat, breathing difficulty during stay in quarantine (ARR = 0.35; 95% CI = 0.20, 0.62) were significantly associated with decreasing anxiety symptoms. In the unadjusted models of stress scores, unable to get sufficient information about the quarantine; have no a plan of what to do after the quarantine; and experienced headache, sore throat and breathing difficulty during stay in the quarantine center were significantly associated with increasing stress scores. In the multivariable model, have no a plan of what to do after the quarantine (ARR = 1.30; 95% CI = 1.09, 1.54) was significantly associated with increasing stress scores. On the other hand, have not experienced headache, sore throat, breathing difficulty during stay in quarantine (ARR = 0.43; 95% CI = 0.28, 0.66) was significantly associated with decreasing stress scores.

*COVID-19 = Coronavirus Disease 2019; CRR = Crude relative risk; ARR = Adjusted relative risk; CI = confidence interval.*

*COVID-19 = Coronavirus Disease 2019; CRR = Crude relative risk; ARR = Adjusted relative risk; CI = confidence interval.*

*COVID-19 = Coronavirus Disease 2019; CRR = Crude relative risk; ARR = Adjusted relative risk; CI = confidence interval.*

## Discussion

In this institution-based cross sectional study among migrant returnees who were in quarantine during the time of COVID-19, we found a very high level of psychological distress. More than half of the participants had depressive symptoms; 22.0% had severe or extremely severe symptoms of depression. Nearly half of the participants had anxiety symptoms, with 26.1% sever or extremely severe anxiety symptoms. More than a third of the participants had stress symptoms, with 13.4% sever or extremely severe stress symptoms.

These findings are consistent with previous studies which showed that the prevalence of psychological distress is higher in the migrant population compared to the native population in countries of destination as well as the population in the countries of origin [19, 42–46]. Qualitative studies conducted among Ethiopian migrant returnees from different Middle East countries showed that migrants experience symptoms of psychological distress as they are exposed to different forms of abuse [2, 13, 23]. A cross-sectional survey conducted among a large sample of Ethiopian migrant returnees found a higher prevalence of symptoms of depression, anxiety and somatization [14]. The prevalence of common mental health problems reported in the current study is found to be higher than reports from prevalence studies conducted in the general population in Ethiopia [47–49]. This might be because participants of this study passed through multiple life challenges such as unexpected deportation, novel mandatory quarantine experience, and facing all COVID-19 related challenges in a relatively brief period of time. However, to the best of our knowledge, there are no published studies on the prevalence of psychological distress in the general population as well as among migrant returnees in Ethiopia during the time of COVID-19 to compare our findings with.

Studies in other countries show that the population level prevalence of psychological distress has significantly increased during the time of the COVID-19 pandemic [25, 50–52]. For instance, a nationwide survey among Chinese people during the time of COVID-19 [25] found that almost 35% of the respondents experienced psychological distress. In a study conducted among the general population in China during the initial stage of the COVID-19 pandemic, 16.5% of respondents reported moderate to severe depressive symptoms; 28.8% of respondents reported moderate to severe anxiety symptoms; and 8.1% reported moderate to severe stress levels [53]. A systematic review and meta-analysis of studies conducted in the general population on the prevalence of stress, anxiety and depression during the COVID-19 pandemic found 29.6, 31.9 and 33.7%, respectively [52]. However, the studies included in the review were from Asia (China, Iran, Iraq, Japan and Nepal), Europe (Spain and Italy) and the UK. There are a few studies which found significant impact of COVID-19 on the mental health of healthcare providers. For instance, a cross-sectional survey in Italy [26] found that general practitioners, who are COVID-19 frontline workers, reported moderate to severe depressive symptoms and very severe anxiety and insomnia. The study further highlighted the urgent need to provide continuity of care for patients at the community level, adequate personal protective equipment to general practitioners and a clear guidance from public health institutions. Experiencing quarantine, fear of infection and uncertainties about the future all contribute to the increased level of psychological distress during the time of COVID-19 pandemic [21]. Hence, COVID-19 pandemic and experiencing mandatory quarantine can be considered as double risk to the mental health of migrant returnees.

In this study gender is found to be an important factor significantly associated with depressive and anxiety symptoms. We found significantly higher prevalence of anxiety and depressive symptoms among women than men. Several previous studies also consistently found that depressive and anxiety symptoms are higher among women than men [54, 55]. This may be associated with biological, socio-cultural and economic factors [55, 56]. Empirical studies indicated that international migration in Ethiopia, particularly to the Middle East, is gendered [3, 8]. Ethiopian migrants to the different Middle East countries are mostly women who have lower level of education [1]; most of them are from rural areas [7] and are disadvantaged in terms of family, economic and socio-cultural factors [23].

We found that fear of being infected with COVID-19, fear of discrimination after the quarantine, and experiencing COVID-19 like symptoms, such as headache, sore throat, and breathing difficulty are significantly associated with increasing depressive, anxiety and stress symptoms. Although studies are scarce in this area, a few previous studies conducted in China [25] and Italy [24] found consistent results with our study. Increased fear at the beginning of the COVID-19 pandemic, particularly among people who were in quarantine, is likely to contribute to distress [52]. People are afraid to stay in quarantine as this was a highly stigmatizing experience [21]. If a person is quarantined, they are considered by other people as infected with the virus; and this was particularly the case during the time of the current study. It is also very likely for a person to worry and get panic when they experience COVID-19 like symptoms, such as headache, sore throat, breathing difficulty and increased level of body temperature.

### Strengths and limitations

We conducted a cross-sectional study with a large sample. We managed to collect quality data with a higher response rate. We are able to study the impact of migration and quarantine on the psychological distress level of returnees in the context of COVID-19. Nevertheless, the study findings need to be interpreted taking the following limitations into account. First, this study is not a community-based study; participants are recruited from quarantine centers. Hence, our sample is not representative and the findings are not generalizable to all migrant returnees. For instance, the participants of this study do not represent those migrant returnees stayed by themselves in hotels in the quarantine period. Second, the study is cross-sectional and significant associations do not rule out reverse causality. Third, the sample for the study is biased towards women; more than 92% of the participants of the study were women. Hence the findings of the study may not be well generalized to men migrant returnees. Lastly, the instrument we used to measure the primary outcomes of the study (depression, anxiety and stress) has not been validated in the Ethiopian socio-cultural context, although it has been used in several previous studies.

## Conclusion and implications

Overall, the study shows that there is a very high prevalence of depressive, anxiety and stress symptoms among migrant returnees who are experiencing quarantine in association with COVID-19. The prevalence of psychological distress is found to be significantly higher among women than men. Quarantine and COVID-19 related factors, including fear of being infected with the virus, fear of discrimination after the quarantine, and experiencing COVID-19 like symptoms were significantly associated with increasing prevalence of depressive, anxiety and stress symptoms.

The findings of this study suggest implementing mass screening of psychological distress in this population for proper intervention and referral. Suitable mental health interventions need to be in place in quarantine centers and for migrant returnees in order to address the huge and double burden of mental health problems in this group. Mental health services need to be integrated with medical and other psychosocial and socioeconomic support services. There may be a few severe cases of mental disorders in quarantine centers, especially among migrant returnees. Hence, effective and efficient referral system should be instituted. Prospective longitudinal studies to determine change in the prevalence and severity levels of psychological distress after quarantine are warranted.

## Supplementary Information

**Additional file 1.** Sociodemographic, migration, quarantine and COVID-19 related characteristics questionnaire.

## Data Availability

This study is part of a larger thematic research project. Data used for this analysis will become available through the project.

## Abbreviations

COVID-19: Coronavirus Disease 2019
DASS21: The 21 item Depression Anxiety and Stress Scale
SPSS: Statistical Packages for the Social Sciences
RR: Crude relative risk
ARR: Adjusted relative risk
CI: Confidence interval
VPRTT: The Office of the Vice President for Research and Technology Transfer
SD: Standard deviation
