# Supplementary material for: Psychological distress among Ethiopian migrant returnees who were in quarantine in the context of COVID-19: institution-based cross-sectional study
Source: BMC Psychiatry. 2021 Aug 25;21:424. doi: 10.1186/s12888-021-03429-2 (PMC8385266; doi:10.1186/s12888-021-03429-2)
Supplement: Supplementary file 1 — Additional file 1. Sociodemographic, migration, quarantine and COVID-19 related characteristics questionnaire. [file 12888_2021_3429_MOESM1_ESM.docx]

Additional file 1: Socio-demographic, migration, quarantine and COVID-19 related characteristics questionnaire

**Addis Ababa University**

**Office of the Vice President for Research and Technology Transfer**

**(Questionnaire to be completed by migrant returnees who are in quarantine)**

**Part I: Socio-demographic, Migration, Quarantine and COVID-19 related characteristics**

Below are questions related to your personal information and experiences related to migration, quarantine and COVID-19. Circle the choice that represents your experience to questions which have alternative responses and write your answers in the space provided to open ended questions.

1. Age (in years) _____________________________________________
2. Gender 1. Female 2. Male
3. Educational level
4. Can’t read and write
5. Can read and write (primary)
6. Secondary education
7. Certificate or Diploma
8. First degree or above
9. Marital status
10. Never married
11. Married
12. Divorced
13. Separated
14. Widowed
15. Your status in the host country before return
16. On job
17. Detention center
18. Prison
19. Unemployed
20. What was your host (destination country)_____________________________________
21. How did you go to the destination country? (Way of migration)
22. Through travel agency
23. Through broker
24. Other, specify---------------------------------------------------------
25. Do you have underlying physical health problem (e.g. heart, lung, asthma, diabetes, blood pressure)?
26. Yes, I have
27. No, I haven’t
28. Do you have underlying mental health problem?
29. Yes, I have
30. No, I haven’t
31. Do you have fear of infection in the quarantine center?
32. Yes, I have
33. No, I haven’t
34. Do you think that staying in quarantine protected you not to transmit the virus to family and community?
35. Yes
36. No
37. Do you think that quarantine limits your activities and social interaction?
38. Yes
39. No
40. Do you think that the overall services in the quarantine center were satisfactory?
41. Yes
42. No
43. Do you know the reason why you are here in quarantine?
44. Yes, I know
45. No, I don’t
46. Do you get sufficient information about the quarantine from the concerned body?
47. Yes
48. No
49. Do you have fear of discrimination after the quarantine?
50. Yes
51. No
52. Can you get support from family and relatives after the quarantine?
53. Yes
54. No
55. Do you have a plan of what to do after the quarantine?
56. Yes
57. No
58. Do you have sufficient amount of money for your living and startup business after the quarantine?
59. Yes, I have
60. No, I have not
61. DO you think that you have adequate knowledge about the mode of transmission and prevention of the Coronavirus?
62. Yes, I have
63. No, I have not
64. Have you experienced headache, sour throat, breathing difficulty during your stay in quarantine?
65. Yes, I have
66. No, I have not
67. Did you have contact with a COVID-19 suspected or infected person or were you exposed to situations before the quarantine?
68. Yes, I did
69. No, I didn’t
